# The p53-targeting human phosphatase hCdc14A interacts with the Cdk1/cyclin B complex and is differentially expressed in human cancers

**DOI:** 10.1186/1476-4598-5-25

**Published:** 2006-06-19

**Authors:** Michelle T Paulsen, Adrienne M Starks, Frederick A Derheimer, Sheela Hanasoge, Liwu Li, Jack E Dixon, Mats Ljungman

**Affiliations:** 1 Department of Radiation Oncology, Division of Radiation & Cancer Biology, University of Michigan Comprehensive Cancer Center, University of Michigan, Ann Arbor, MI 48109, USA; 2 Department of Biology, Virginia Tech, Blacksburg, VA 24061-0406, USA; 3 Departments of Pharmacology, Cellular & Molecular Medicine and Chemistry & Biochemistry, University of California, San Diego, La Jolla, CA 92093-0636, USA; 4 Department of Environmental Health Sciences, School of Public Health, University of Michigan, Ann Arbor, MI 48109, USA

## Abstract

**Background:**

The evolutionary conserved cyclin-dependent kinase phosphatase hCdc14A has been shown to play potential roles in the regulation of mitotic exit and in the centrosome duplication cycle. We have recently shown that hCdc14A also can interact with the tumor suppressor p53 both *in vitro *and *in vivo *and specifically dephosphorylates the ser315 site of p53 *in vitro*. In this study we developed antibodies against hCdc14A to investigate the expression and regulation of hCdc14A in human tissues and cancer cells.

**Results:**

We show that hCdc14A is differentially expressed in human tissues and in 75 cancer cell lines examined. Treatments with the histone deacetylase inhibitor TSA, the demethylating agent 5-aza-2'-deoxycytodine or the proteasome inhibitor MG132 significantly induced expression of hCdc14A in cell lines expressing low or undetectable levels of hCdc14A. There was a strong bias for low expression of hCdc14A in cancer cell lines harboring wild-type p53, suggesting that high Cdc14A expression is not compatible with wild-type p53 expression. We present evidence for a role for hCdc14A in the dephosphorylation of the ser315 site of p53 *in vivo *and that hCdc14A forms a complex with Cdk1/cyclin B during interphase but not during mitosis.

**Conclusion:**

Our results that hCdc14A is differentially expressed in human cancer cells and that hCdc14A can interact with both p53 and the Cdk1/cyclin B complex may implicate that dysregulation of hCdc14A expression may play a role in carcinogenesis.

## Background

The protein phosphatase Cdc14 has been shown to be essential for survival and critical for the resolution of mitosis in *Saccharomyces cerevisiae *and *Caenorhabditis elegans *[1-3]. It is thought that Cdc14 in *S. Cerevisiae *is regulated both spatially and temporally by cyclin B-Cdk and the mitotic-exit network (MEN), which releases Cdc14 from the inhibitory RENT complex in the nucleolus in late anaphase [4-6]. When released, Cdc14 inactivates cyclin B-Cdk while activating the anaphase promoting complex (APC) that targets the destruction of mitosis-specific cyclins [7,8]. In the fission yeast *S. pombe*, the Cdc14 homologue Flp1/Clp1 appears to be differently regulated and may have distinct functions from its homologue in *S. cerevisiae *[9]. Flp1/Clp1 is not an essential gene in *S. pombe *and is thought to act in the G_2 _phase of the cell cycle, during exit from mitosis and cytokinesis by regulating the Cdc25 phosphatase and the wee1 kinase [10-15].

We previously cloned two human orthologs of the yeast Cdc14 gene, hCdc14A and hCdc14B [16]. The hCdc14A gene is located on band 1p21 on chromosome 1 and consists of 16 exons spread over 170 kbp of DNA (genbank). Three different splice variants of hCdc14A have been found but it is not known whether all of them are translated into proteins. The hCdc14A has dual specific phosphatase activity and it can rescue Cdc14 mutants in *S. cerevisiae *and *S. pombe *[16,17] suggesting that human Cdc14A and yeast Cdc14 play similar functional roles [18]. In further support of similarities in function is the finding that hCdc14A can, like Cdc14 in *S. cerevisiae*, activate the anaphase promoting complex (APC) in late anaphase by dephosphorylating the APC co-factor Cdh1 [19] and dephosphorylate substrates of cyclin-dependent kinases [17,20,21]. However, hCdc14A appears to be associated with centrosomes [19,21,22] while the yeast Cdc14 is predominantly localized to nucleoli [4,5]. Forced over expression of hCdc14A has been shown to result in premature centrosome splitting and mitotic spindle and chromosome segregation defects while reduced expression using siRNA techniques results in failure of both centrosome separation and cytokinesis [21,22].

In addition to its potential role in the regulation of mitotic exit and in the centrosome duplication cycle [19,21,22], we have recently shown that hCdc14A can interact with the tumor suppressor p53 both *in vitro *and *in vivo *and specifically dephosphorylates the ser315 site of p53 *in vitro *[20]. This site can be phosphorylated by Cdk2/cyclin A and Cdk1/cyclin B *in vitro *[23] and by aurora kinase A in cells [24] suggesting that the hCdc14A phosphatase may act as a Cdk and aurora kinase antagonist [20,21,25].

A previous study investigating the frequency of mutations in the *hCdc14A *gene in human cancer cell lines found that this gene is rarely mutated in human cancers [26]. However, the findings that manipulations of the protein levels of hCdc14A result in impaired chromosome segregation and aborted cytokinesis in human cells suggests that dysregulation of protein expression of hCdc14A may contribute to carcinogenesis [21,22]. To investigate the expression levels of the hCdc14A protein in human cancer cell lines we raised monoclonal antibodies against both the N- and C-terminal domains of hCdc14A. Our results show that hCdc14A is differentially expressed in human cancer cell lines and that the low expression of hCdc14A could be enhanced by blocking DNA methylation, histone deacetylation or proteasome activity. Our results also show that high expression of hCdc14A was rarely found in tumors expressing wild-type p53 suggesting that over expression of hCdc14A is not compatible with wild-type p53 function. Finally, we found that hCdc14A interacts with Cdc2/cyclin B during interphase but not during mitosis suggesting that hCdc14A may ensure that Cdk1/cyclin B activity is held in check until it is needed during mitosis.

## Results

### Expression profile of hCdc14A in human tissue cells and cancer cell lines

**Figure 1:**
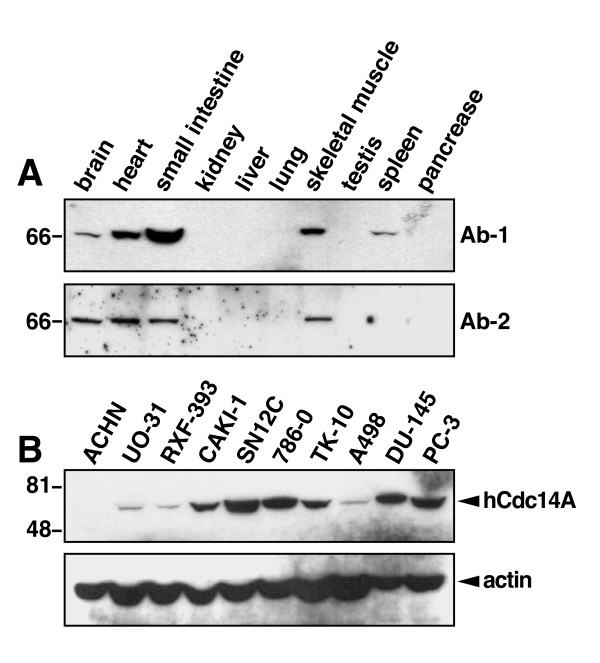
Differential expression of hCdc14A protein in human tissues and human cancer cell lines. **A) **The two different anti-hCdc14A antibodies Ab-1 (top) and Ab-2 (bottom) were used to analyze the amount of hCdc14A protein expression in different tissues using two independent INSTA-blot membranes (IMGENEX) containing human tissue lysates. Both antibodies detect a major band of 66–67 kDa size and revealed a differential expression of hCdc14A in human tissues. The blots were then stained with Coomassie Blue stain to verify even loading of proteins in the different lanes (data not shown). **B) **Equal amounts of protein from each cancer cell line were loaded in each lane and the amount of hCdc14A was detected by Western blot using the Ab-2 antibody. Actin protein levels were used as loading/transfer controls. A tabulation of the results from this figure and from other experiments including other cell lines can be found in Table I.

**Figure 4:**
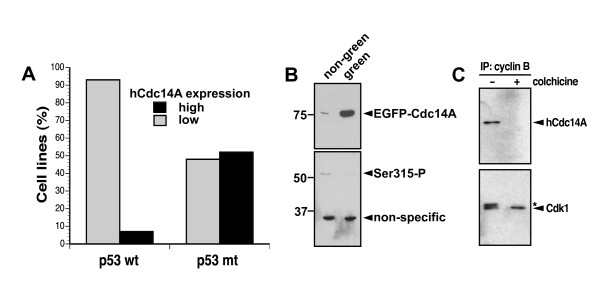
Potential role of hCdc14A in regulating p53 and Cdk1/cyclin B. **A) **The results in Table I were used to compare the relationship between the expression level of hCdc14A and p53 status of the cell lines. The cell lines CAK-1 and SF-539 were excluded in the analysis since they have been shown to not arrest in G1 following exposure to ionizing radiation [35] and the HCT116 cells were excluded because of conflicting results of hCdc14A expression between two different sources of this cell line. **B) **COS cells were transiently transfected with the pEGFP-hCdc14A vector and 24 hours later the GFP positive cells were sorted from the GFP negative cells and expression of hCdc14A and phosphorylation of the ser315 site of p53 was assessed using immunoblotting using anti-hCdc14A (Ab-2) and anti-ser315 phospho-specific p53 antibodies. **C) **HCT116 cells were mock treated or treated with the microtubular inhibitor colchicine for 18 hours. Cells were then lysed and cyclin B was immunoprecipitated using specific antibodies. The proteins recovered were then denatured and separated using SDS-PAGE. Anti-hCdc14A and anti-Cdk1 antibodies were then used in immunoblotting. The star denotes the phosphorylated inactive form of Cdk1.

It has been reported that alternative splicing of the hCdc14A gene can give rise to three transcript variants encoding distinct hCdc14A isoforms (LocusLink, NCBI). To assess hCdc14A protein expression and determine which of these isoforms are translated into proteins in cells, we raised mouse monoclonal antibodies against polypeptides representing sequences from both the N-terminus (Ab-1) and C-terminus (Ab-2) of hCdc14A (see Materials and Methods). Using these monoclonal antibodies and Western blot we first explored the expression of Cdc14A in human tissues. We incubated Ab-1 or Ab-2 anti-hCdc14A monoclonal antibodies with two independent INSTA-blot membranes (IMGENEX) containing pre-blotted proteins isolated from different human tissues. It was found that both the Ab-1 and Ab-2 anti-hCdc14A antibodies detect a single band of about 66–67 kDa in size (Figure 1A). This value corresponds well to the size of isoform 1 of hCdc14A, which consists of 594 amino acids with a predicted molecular weight of 66.6 kDa. Thus, it appears that only isoform 1 of hCdc14 is successfully translated into a protein product in human cells and that both the Ab-1 and the Ab-2 antibodies work well to detect this isoform of hCdc14A by immunoblotting. Furthermore, the specificity of these antibodies was verified by detection of a single band in COS cells over expressing hCdc14A-GFP (see Figure 4B). Using these antibodies it was found that hCdc14A is differentially expressed in human tissues with high protein expression in brain, heart, small intestine and skeletal muscle, moderate expression in spleen and low or undetectable expression in kidney, liver, lung, testis and pancreas. Differential expression of hCdc14A mRNA in human tissues has previously been reported [16].

We next examined the expression of hCdc14A protein in various human cancer cell lines either grown in the lab or obtained as cell pellets from NCI. Western blot using the hCdc14A (Ab-2) antibody revealed differential expression of hCdc14A in human cancer cell lines (Figure 1B and Table 1). Low hCdc14A expression was especially common in melanoma and neuroblastoma cancer cell lines. However, cell lines from other cancer sites showed differential expression even within a specific tumor type. It can be noted that the HCT116 cells obtained as a cell pellet showed low hCdc14A expression while the two HCT116 cell lines grown in our lab showed high expression. The reason for this discrepancy is not known. Furthermore, the MDA-MB-436 breast cancer cell line, which was previously reported to only have one wild-type hCdc14A allele, which reportedly is silent [26], expressed a low but detectable amount of hCdc14A protein.

**Table I:** Expression of Cdc14A protein in human cancer cell lines as determined by Western blot using anti-hCdc14A antibodies (Ab-2).

**Cell lines**	**Tumor type**	**Cdc14A expression**	**P53 status**
MCF-7	breast	high	wt
MCF7/ADR-RES		very high	mt
MDA-MB-231		high	mt
MDA-MB-435		not detected	mt
HS 578T		not detected	mt
T-47D		low	mt
BT-549		high	?
MCF-7*		high	wt
MCF-10*		not detected	wt
SUM-44*		low	?
SUM-468*		high	mt
MDA-MB-436*		low	mt

A549/ATCC	lung	low	wt
NCI-H460		not detected	wt
NCI-H23		not detected	mt
NCI-H322M		not detected	mt
EKVX		not detected	mt
NCI-H226		high	mt
HCI-H522		not detected	mt
HOP-62		high	mt
HOP-92		high	mt

HCT-116	colon	low	wt
HCT-15		low	wt/mt
HT29		high	mt
HCC2998		high	mt
SW-620		not detected	mt
COLO 205		low	mt
KM12		low	mt
HCT-116*		high	wt
HCT-116 p53-/-*		high	null
RKO*		low	wt
HT29*		high	mt

ACHN	kidney	not detected	wt
UO-31		low	wt
RXF-393		low	mt
CAKI-1		high	wt
SN12C		high	mt
786-0		high	mt
TK-10		high	mt
A498		low	wt

OVCAR-4	ovary	not detected	wt
IGROV1		low	wt
OVCAR-3		high	mt
OVCAR-8		very high	mt
OVCAR-5		high	mt
SK-OV-3		low	mt

SF-539	CNS	high	wt
SF-295		high	mt
SF-268		low	mt
SNB-75		high	mt
SNB-19		not detected	mt
U251		high	mt

MOLT-4	leukemia	not detected	wt
SR		high	wt
RPMI-8226		high	mt
HL-60(TB)		not detected	mt
K-562		low	mt
CCRF – CEM		not detected	mt

MALME-3M	melanoma	low	wt
SK-MEL-5		not detected	wt
UACC-62		not detected	wt
LOX IMVI		low	wt
SK-MEL-28		not detected	mt
M14		not detected	mt
UACC-257		not detected	wt
SK-MEL-2		not detected	wt

DU-145	prostate	high	mt
PC-3		high	mt
PC-3*		low	mt
LNCaP*		low	wt

J82*	bladder	very high	mt

SK-N-SH*	neroblastoma	low	wt
Sk-N-MC*		low	wt
IMR32*		not detected	wt
SHSY-SY*		low	wt

Normal fibroblasts (NF)*	others	low	wt
COS*		not detected	wt

* cell lines grown in the lab. All the other samples were from NCI.

### Induction of hCdc14A by 5Aza-dC and TSA

Since a previous mutational analysis of the hCdc14A locus in a large panel of human tumor cell lines did not reveal an abundance of deletions or mutations [26], we thought that the lack of expression of hCdc14A in many of the cancer cell lines tested may be due to DNA hypermethylation and/or histone hypoacetylation. To test this possibility we treated some of our low hCdc14A-expressing cells with the demethylation agent 5Aza-dC and the histone deacetylation inhibitor TSA. It has been shown that these agents can reactivate the expression of genes with DNA hypermethylation [27] or histone hypoacetylation [28].

**Figure 2:**
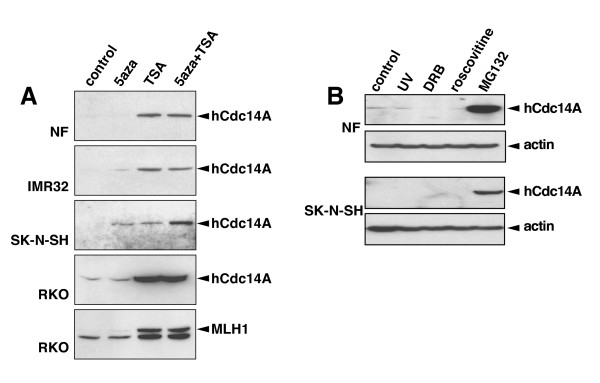
Up-regulation of hCdc14A protein expression following treatment with 5aza-dC and/or TSA or MG132. **A) **Cells were treated with 5aza-dC and TSA alone or in combination and the levels of hCdc14A and MLH1 were analyzed using Western blot and anti-hCdc14A (Ab-2) or anti-MLH1 antibodies. hCdc14A was up-regulated by both 5aza-dC and TSA in the two neuroblastoma cell lines IMR32 and SK-N-SH while in normal fibroblasts and RKO cells hCdc14A levels only responded to TSA. **B) **Normal fibroblasts (top) or SK-N-SH neuroblastoma cells (bottom) were either UV-irradiated (20 J/m^2^) or treated for 16 hours with the transcription inhibitor DRB (100 μM), the Cdk and transcription inhibitor roscovitine (50 μM) or the proteasome inhibitor MG132 (10 μM) before the cells were harvested and hCdc14A protein levels analyzed on Western blot using anti-hCdc14A (Ab-1) or anti-actin antibodies (loading control).

In normal fibroblasts and RKO colon cancer cells, treatment with the demethylating agent 5Aza-dC for 48 hours did not induce expression of hCdc14A (Figure 2A). As a positive control for demethylation we included a blot for the MLH1 protein in RKO cells which gene is known to be silenced by hypermethylation [27]. We did observed some re-activation of MLH1 protein expression using 5Aza-dC in the RKO cells, although the expression level was not impressive. In the neuroblastoma cell lines IMR32 and SK-N-SH, the 5Aza-dC treatment resulted in a detectable increase in hCdc14A protein expression suggesting that the hCdc14A gene may be silenced by hypermethylation in these cells. When cells were treated with the histone deacetylase inhibitor TSA, we observed a substantial increase in hCdc14A protein expression in all cell lines tested. The expression of the MLH1 protein in RKO cells was also dramatically increased by TSA. Finally, the combination of 5Aza-dC and TSA resulted in an additive increase of hCdc14A protein expression only in the SK-N-SH cell line. Taken together, these results suggest that the hCdc14A gene may be epigenetically silenced by DNA hypermethylation and/or histone hypoacetylation in some human cancer cell lines.

### Proteasome inhibition results in increased Cdc14A expression

We next investigated whether hCdc14A could be induced by cellular stresses such as UV light, the transcription inhibitor DRB (5,6-dichloro-1-b-D-ribofuranosylbenzimidazole) [29,30], the Cdk and transcription inhibitor roscovitine {2-(1-ethyl-2-hydroxyethylamino)-6-benzylamino-9-isopropylpurine} [31-33] or the proteasome inhibitor MG132 [34]. No induction of hCdc14A was observed in normal fibroblasts or SK-N-SH neuroblastoma cell following exposure to UV light, DRB or roscovitine (Figure 2B). However, large amounts of hCdc14A accumulated in both cell types following treatment with the proteasome inhibitor MG132 suggesting that hCdc14A may normally be subject to proteasome-mediated degradation.

**Figure 3:**
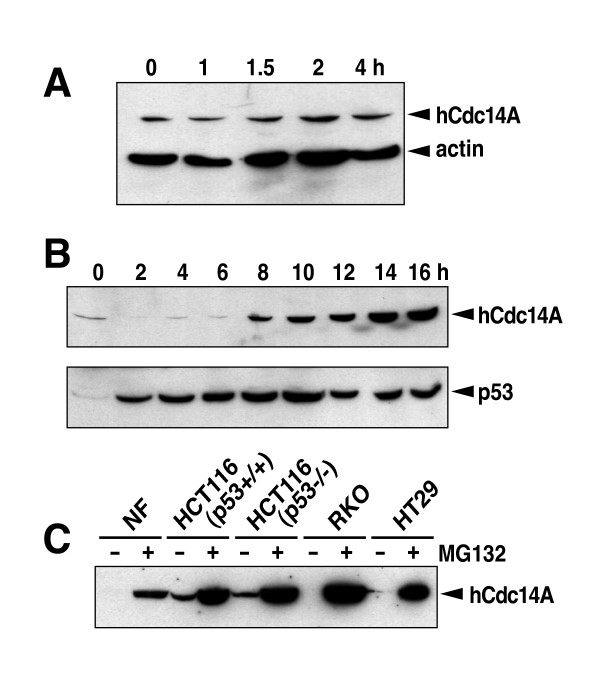
Despite being a stable protein, Cdc14A expression can be significantly increased by incubation with the proteasome inhibitor MG132. **A) **HCT116 colon cancer cells were incubated for different periods of time in the presence of the protein synthesis inhibitor cycloheximide (10 μg/ml) followed by Western blot with anti-hCdc14A (Ab-1) and anti-actin antibodies. **B) **Human diploid fibroblasts were incubated for different periods of time with the proteasome-inhibitor MG132 (10 μM) followed by western blot with anti-hCdc14A (Ab-1) or anti-p53 antibodies. **C) **Cells were mock treated or incubated for 16 hours in the presence of MG132 (10 μM) followed by Western blot using anti-hCdc14A (Ab-1) antibodies.

To investigate whether the Cdc14A phosphatase under normal conditions is subjected to proteasome-mediated degradation, we measured the Cdc14A protein level in HCT116 cells incubated for different time periods in the presence of the protein synthesis inhibitor cycloheximide. Surprisingly, we found that the Cdc14A protein is under non-stressed condition a stable protein with no detectable protein turnover within 4 hours (Figure 3A). We next investigated the time course for the accumulation of hCdc14A proteins following proteasome inhibition with MG132. It was found that the expression of the Cdc14A protein sharply increased after about 8 hours (Figure 3B). The kinetics of hCdc14A protein accumulation is very different from the kinetics of p53 protein accumulation following proteasome inhibition. Since the p53 protein is normally rapidly degraded in a proteasome-dependent manner, the inhibition of proteasome activity by MG132 rapidly results in the stabilization and accumulation of p53 (Figure 3B, lower blot). Thus, even though hCdc14A dramatically accumulates following proteasome inhibition, our results do not suggest that the hCdc14A protein is rapidly turning over in a proteasome-dependent manner.

Since MG132 incubation led to the induction of p53 prior to the induction of hCdc14A, we thought that perhaps p53 was mediating the expression of hCdc14A. To investigate whether the induction of hCdc14A by MG132 was p53-dependent, we compared the induction of hCdc14A following MG132 incubation in isogenic HCT116 cell lines differing only in their p53 status. It was found that hCdc14A levels increased to the same extent following MG132 treatment in both cell lines (Figure 3C). Furthermore, hCdc14A protein levels were significantly increased also in the p53 mutant cell line HT29. In fact, MG132 substantially increased hCdc14A expression irrespective of p53 status in all cell lines tested.

### Inverse correlation between hCdc14A expression and p53 wild-type status

In table 1, the p53 status of each of the cell lines used in this study are listed as well as their relative hCdc14A protein expression level. It can be noticed that most of the p53 wild-type containing cells expressed little or undetectable levels of hCdc14A. Only five of the 29 cell lines (17%) expressing non-mutant p53 expressed high levels of hCdc14A. Two of these five cell lines (CAK-1 and SF-539) have been shown to lack a G_1 _cell cycle arrest following exposure to ionizing radiation [35] suggesting that the p53 response pathway in these cells may be abrogated by an alternative mechanism than p53 mutation. Excluding these two wtp53 cell lines with abrogated p53 responses and the HCT116 cell line, with which we obtained conflicting results on hCdc14A expression levels between two sources, only 2 out of 26 wt p53 cell lines (8%) expressed high levels of hCdc14A (Figure 4A). These were the breast cancer cell line MCF-7 and the leukemia cell line SR. In contrast to the wt p53 expressing cells, 51% of cells with mutant p53 expressed high levels of hCdc14A. These results suggest that over expression of hCdc14A in cells with wild-type p53 may not be tolerated perhaps because these tumor cells may undergo a p53-mediated cell death.

### Overexpression of hCdc14A results in dephosphorylation of the ser315 site of p53

We have previously shown that hCdc14A can bind to and dephosphorylate the ser315 site of p53 *in vitro *[20]. To investigate whether hCdc14A is capable of dephosphorylating the ser315 site of p53 *in vivo*, we transfected COS cells with a vector expressing EGFP-hCdc14A [20]. COS cells do not normally express detectable levels of hCdc14 (Table 1). After a 48-hour expression time following transfection, cells were sorted according to EGFP expression using a FACS cell sorter and cell lysates were subjected to Western blotting. As expected, the expression of EGFP-hCdc14A was much higher in the cells sorted for green fluorescence compared to the non-green sorted cells (Figure 4B, top panel). This confirmed that the FACS sorting was fairly clean and that our antibodies specifically detect hCdc14A. Using a ser315 phospho-specific p53 antibody (Cell Signaling), phosphorylation of the ser315 site of p53 could be detected in the non-green cells but not in the cells expressing high levels of hCdc14A-GFP (Figure 4B, bottom panel). A non-specific band produced by this antibody demonstrates even loading of the samples. These results show that over expression of hCdc14A results in loss of ser315 phosphorylation of p53 suggesting that hCdc14A dephosphorylates the ser315 site of p53 *in vivo *as we have previously shown *in vitro *[20].

### hCdc14A interacts with the Cdk1/cyclin B complex in interphase but not in mitosis

The hCdc14A and hCdc14B phosphatases have been shown to antagonize Cdks by dephosphorylating Cdk substrates [17,21]. Since Cdk1/cyclin B complexes co-localize with hCdc14A to centrosomes [36,37], it is possible that hCdc14A and Cdk1/cyclin B associate with each other. To test this hypothesis, we immunoprecipitated cyclin B with anti-cyclin B antibodies and found that hCdc14A co-immunoprecipitated with cyclin B in asynchronized cells (Figure 4C). In contrast, when cyclin B was immunoprecipitated from cells arrested in mitosis following an 18-hour treatment with colchicine, no hCdc14A was found to co-immunoprecipitate with cyclin B. As expected Cdk1 was co-immunoprecipitated with cyclin B from both asynchronized and colchicine-treated cells. These results suggest that hCdc14A forms a complex with Cdk1/cyclin B during interphase but hCdc14A separates from Cdk1/cyclin B when cells enter mitosis.

## Discussion

The hCdc14A phosphatase has recently been shown to bind to and dephosphorylate the tumor suppressor p53 [20] as well as participate in cell cycle regulation [19,38]. Thus, dysregulation of hCdc14A may potentially play a role in carcinogenesis. However, the frequency of mutations found in the *hcdc14A *gene in tumor cell lines is fairly low [26]. Using two different monoclonal anti-hCdc14A antibodies we here show that the hCdc14A phosphatase is differentially expressed in human cancer cell lines (Table 1). In melanoma and neuroblastoma cell lines hCdc14A expression was found to be very low or not detectable while its expression varied dramatically between different cell lines from other tumor types. When the protein expression of hCdc14A in the different cancer cell lines was compared to the p53 status of the lines, a strong bias against high hCdc14A expression was observed in wild-type but not mutant p53-expressing cells (Figure 4A). The strong bias for low expression of hCdc14A in cancer cells with wild-type p53 status compared to cancer cells with mutant p53 suggests to us that there is a strong selection process against cells expressing high levels of hCdc14A in the context of functional p53. It is possible that high hCdc14A expression may activate p53 leading to cell cycle arrest or apoptosis.

We have previously shown that hCdc14A and hCdc14B can dephosphorylate the ser315 site of p53 *in vitro*. Here we show evidence of a similar role for hCdc14A in dephosphorylaing the ser315 site of p53 *in vivo*. The ser315 site of p53 can be phosphorylated by Cdk2/cyclin A and Cdk1/cyclin B *in vitro *[23] and by aurora kinase A in cells [24]. The ser315 site of p53 has been shown to be crucial for the binding of p53 to unduplicated centrosomes which is important in the regulation of the centrosome duplication cycle [39-41]. Both hCdc14A and hCdc14B can rescue Cdc14 defects in yeast cells [16,17], suggesting that they are functional homologues. However, while hCdc14A localizes to centrosomes the hCdc14B phosphatase localizes to nucleoli suggesting that the two phosphatases may have different sets of substrates and therefore may serve distinct functions in cells.

The association of hCdc14A to centrosomes [19,21,22,38] and the fact that it can interact with and dephosphorylate p53 both *in vitro *and *in vivo *[20] suggest that hCdc14A may perhaps regulate p53 function. Moreover, its centrosomal localization puts it in close proximity to a number of other cell cycle kinases and their substrates such as Cdk1 [42], Cdk2 [43,44], Plk1 [45], aurora kinases [46-48] and BRCA1 [49]. Our results showing that hCdc14A is in a complex with Cdk1 and cyclin B during interphase but not during mitosis (Figure 4C) suggest that hCdc14A may ensure that the activity of the Cdk1/cyclin B complex is suppressed by hCdc14A during interphase but not during mitosis.

Hypermethylation and histone hypoacetylation of promoter regions leading to inactivation of tumor suppressor genes is a common event during carcinogenesis [27,50,51]. The low expression of hCdc14A in some of the cell lines tested appeared to be due to hypermethylation and/or histone hypoacetylation of the hCdc14A promoter since hCdc14A expression was enhanced by treatment with the demethylating agent 5aza-dC and/or the histone deacetylase inhibitor TSA. Our finding that the proteasome inhibitor MG132 dramatically increased the hCdc14A protein levels in all cell lines tested was surprising in light of the apparent intrinsic stability of the hCdc14A protein. The mechanism for this enhancement is not understood but could involve the stabilization of a labile protein involved in the positive regulation of hCdc14A expression. Since MG132 treatment lead to nuclear accumulation of p53 that preceded Cdc14A accumulation by about 6 hours, we first thought that p53 may be involved in the induction of Cdc14A following proteasome inhibition. However, when cells lacking functional p53 were challenged with MG132 we observed a similar increase in Cdc14A protein levels as in cells harboring wild-type p53 ruling out a possible regulation of hCdc14A by p53.

**Figure 5:**
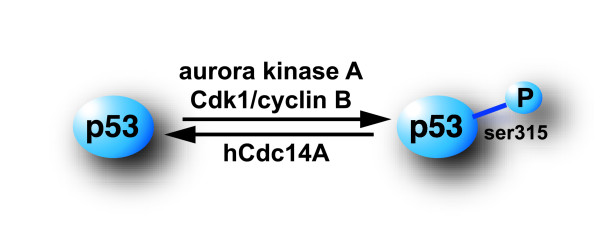
Model outlining the role of hCdc14A as a p53-targeting phosphatase. The ser315 site of p53 can be phosphorylated by aurora kinase A and the Cdk1/cyclin B complex. In this study we show evidence that hCdc14A can antagonize these kinases by dephosphorylating the ser315 site in cells. In addition, by interacting with Cdk1/cyclin B during interphase, hCdc14A may further antagonize Cdk1/cyclin B-mediated phosphorylation until cells enter mitosis.

It has been shown that the PP1 phosphatase can be found associated with the centrosomes [52] and that it is involved in a feedback regulation of the aurora kinase A at the centrosome throughout the cell cycle [53]. This finding is giving precedence for a scenario in which protein phosphatases may be able to antagonize protein kinases at the centrosome. Since we here show that hCdc14 can dephosphorylate the ser315 site of p53 in cells and interact with Cdk1/cyclin B during interphase (Fig. 5), it is tempting to speculate that hCdc14A may play a role in suppressing carcinogenesis by regulating p53 and act as an antagonist of Cdk kinases.

## Conclusion

The strong bias against over expression of hCdc14 in human tumor cell lines expressing wild-type p53 suggests that high expression of hCdc14A may be selected against by p53-mediated cell cycle arrest or cell death. Conversely, over expression of hCdc14A may set up a strong selection for inactivation of p53 function in tumor cells. Together with previous findings that hCdc14A regulates centrosome function and cytokenesis, our findings that hCdc14A may regulate p53 and Cdk1/cyclin B suggest that dysregulation of hCdc14A may play an important role in carcinogenesis.

## Methods

### Cell cultures

Human neonatal diploid fibroblasts, RKO colon cancer cells and the bladder cancer cell line J82 were grown in MEM supplemented with 10% fetal bovine serum (FBS), 2× vitamins, 2× amino acids and 1× antibiotics. RPMI supplemented with 10% FBS and 1× antibiotics was used for the breast cancer cell lines MCF-7, MCF-10, SUM-44, SUM-102 and MDA-468, the colon cancer cell lines HT29, HCT116 and HCT116 (p53-/-), the prostate cancer cell lines PC-3 and LNCaP. The neuroblastoma cell lines SK-N-SH, SCN-MC, IMR32 and SHSY-SY were grown in MEM supplemented with 10% FBS and 2× amino acids and 1 mM sodium pyruvate. The COS cells were grown in DMEM supplemented with 10% FBS and 1× antibiotics. Cells were seeded two days prior to experiments or harvest for Western blot. Richard F. Camalier, NCI, Frederick, MD kindly supplied the cell pellets of the NCI 59 cancer cell lines.

### Irradiation and drug treatments

For some experiments, 5-aza-2'-deoxycytidine (5aza-dC) and/or trichostatin A (TSA) were used in concentrations of 100 nM and 300 nM, respectively, as previously described [27]. For UV-irradiation, cells were irradiated with 20 J/m^2 ^of UV light (254 nm) at room temperature at a fluency of about 0.6 J/m^2^/sec (UVX radiometer, UVP, Inc, Upland, CA). MG132 (corbobenzoxy-L-leucyl-L-leucyl-L-leucinal) (Calbiochem, La Jolla, CA) was added from a 10 mM stock solution in DMSO to a final concentration of 10 μM. DRB (5,6-dichloro-1-b-D-ribofuranosylbenzimidazole) (Sigma, St. Louis, MI) was used in a 100 μM concentration from a 50 mM stock in ethanol and roscovitine (Calbiochem, La Jolla, CA) was added from a 10 mM stock solution in DMSO to a final concentration of 50 μM.

### Production of anti-Cdc14A monoclonal antibodies

The peptide sequences RPKSTVNTHYFSIDEEL (Ab-1), which spans amino acids 13–29 of hCdc14A according to Li et al. [16] and DPENKKTSSSSK (Ab-2), which spans the amino acids 480–491, were synthesized at the Protein and Carbohydrate Structure Facility in the Biopolymer Core, University of Michigan Medical School. These peptides were purified before being conjugated to KLH and used to immunize mice (performed at the University of Michigan Hybridoma Facility). Blood sera from the immunized mice were screened by ELISA using the KLH-Cdc14A peptides as a positive control and KLH-p53 peptides as a negative control. The spleen cells from one of the immunized mice in each set were then used to produce hybridoma cells that were clonally expanded and further screened by ELISA.

### Immunoblotting

Cells were prepared for western blots as previously described [54,55]. The supernatant from the final clones selected producing monoclonal anti-Cdc14A antibodies Ab-1 and Ab-2 were used in a 1:5 and a 1:10 dilution, respectively. Both Ab-1 (N-terminus) and Ab-2 (C-terminus) recognized a single major band on Western blots in the size range of 66–67 kDa which is the expected size for the 594 amino acids protein (66.6 m.w.). In addition to the major band, the antibody detected a minor band at about 20 kDa. While the intensity of the major band varied significantly between different cell lines, the 20-kDa band was invariant. For the examination of Cdc14A expression in human tissues, an INSTA-blot (IMB-103, IMGENEX, San Diego, CA) was used. Similar protein concentrations in the different lanes on the INSTA-blot were confirmed by using the Ponceau S. protein stain. For Western blot of the Cdc14A from cancer cell lines, equal amounts of proteins were loaded onto 10% polyacrylamide/SDS gels and after blotting, equal transfer of proteins was confirmed by staining the membranes with anti-actin antibodies (Sigma Chemical Co., St Louis, MO) and Coomassie Blue stain. Other antibodies used for Western blot were anti-MLH1 (BD Biosciences Pharmingen, San Diego, CA) anti-p53 (Ab-2, Oncogene Research Products, Boston MA) and anti-ser315 phospho-specific p53 antibodies (Cell Signaling).

### Transient transfection and FACS sorting

Cells were transiently transfected with the pEGFP-hCdc14A vector [16] using the FuGENE6 transfection agent according to description of the manufacturer (Boehringer Mannheim, Mannheim, Germany). Cells were then incubated for 24 hours before the cells were trypsinized and sorted according to their expression of green fluorescence using FACS flow sorting. The two fractions were then prepared for immunoblotting

### Immunoprecipitation

HCT116 cells were mock treated or incubated with colchicine (0.5 μg/ml) for 18 hours before the cells were collected and cyclin B was immunoprecipitated using anti-cyclin B1 antibodies (Santa Cruz) as previously described [56]. The immunoprecipitates were then run on 10% SDS/PAGE and proteins were visualized as described for western blots using our monoclonal anti-hCDC14A antibodies (Ab-2) or anti-Cdk1 antibodies (Santa Cruz).

## Competing interests

The author(s) declare that they have no competing interests.

## Authors' contributions

MTP developed the antibodies used in this study and performed most of the experiments presented. AMS helped with estimation of the levels of hCdc14a in tumor samples and FAD and SH performed experiments related to ser315 phosphorylation of p53. LL and JED provided key reagents for the experiments and gave intellectual guidance during the project. ML has designed and directed the laboratory work, interpreted the data and wrote the manuscript.
